# So, you want to get into “total-body” PET/CT scanning? An installation guide for beginners!

**DOI:** 10.1186/s40644-023-00542-1

**Published:** 2023-04-05

**Authors:** Rodney J. Hicks

**Affiliations:** 1 Department of Medicine, St Vincent’s Hospital, The University of Melbourne, Fitzroy, Australia; 2 Department of Medicine, Alfred Hospital Central Medical School, Monash University, Prahran, Australia

**Keywords:** Positron emission tomography, PET, Extended field of view, Total-body PET/CT, Precision medicine

## Abstract

“Total-body” and ultra-extended field-of-view PET/CT scanners are now available commercially with great enthusiasm for their potential in both streamlining clinical practice and providing unique research opportunities. Accordingly, many groups are rushing to implement this technology. For early adopters, the challenges of these systems compared with more standard PET/CT systems have been significant. In this guide, aspects that need to be considered in planning installation of one of these scanners are discussed. These include financing, space, structural engineering, power supply, chilled water and environmental controls to manage heat loads, IT infrastructure and data storage, radiation safety and radiopharmaceutical procurement, staffing levels, patient handling logistics and imaging protocol redesign to leverage the superior sensitivity of these scanners, and marketing. It is a daunting but worthwhile endeavor in the author’s opinion but needs a great team and the ability to bring in the appropriate expertise at the appropriate time.

“Every once in a while, a new technology, an old problem, and a big idea turn into an innovation.”

- Dean Kamen, engineer, businessman and inventor of the Segway vehicle

Everyone loves “new” technology, even when the development involves increments on prior discoveries and advancements that have occurred over many decades. For positron emission tomography/computed tomography (PET/CT), it all started with the application of the mathematical method for filtered back-projection that David Kuhl used in initial experiments in tomographic imaging [1] to X-ray reconstruction of CT images by Gordon Hounsfield and the innovation of the ring configuration PET scanner, which was first developed by the Ter Poggossian group [2] using the coincidence counting of annihilation photons of positron emitting isotopes method that had previously been pioneered by Gordon Brownell’s group in Boston. David Townsend, Thomas Beyer, and their team then combined both instruments into a hybrid PET/CT device [3]. This technology has now effectively replaced stand-alone PET. Three-dimensional, septa-less reconstruction, iterative reconstruction methods, detector blocks capable of time-of-flight measurements and replacement of photomultiplier tubes with solid state processors are amongst a range of innovations that have played a role in dramatically improving the performance of modern PET/CT devices. The next big idea was to dramatically extend the axial field-of-view of such scanners. This was first implemented in the standard ring-configuration by Simon Cherry, Ramsey Badawi and their team at the University of California at Davis [4] and made available commercially as the µExplorer (United Imaging, China). The developers coined the term “total-body” PET/CT as an alternative to “whole-body” PET/CT, which is the method of moving patients through a restricted field-of-view (FOV) detector array, which was developed by Magnus Dahlbom and co-workers [5].

In many clinical situations, “whole-body” PET/CT is a misnomer as many studies are acquired excluding the legs, as these are relatively infrequent sites of metastatic disease for the cancers which dominate oncological practice. Reduced acquisition times can be realized by omitting imaging of the legs, increasing throughput on these valuable scanners. When demand for clinical PET studies is high, the incremental diagnostic information obtained by including the legs must be balanced against the opportunity to scan more patients. Continuous motion through the scanner with reduced acquisition time over the legs can reduce the impost of true whole-body imaging [6]. Limited region scanning is also sometimes done of the brain and heart but less often for other major organs. The vast majority of scans in routine clinical practice are now acquired as a delayed static image and dynamic imaging is rarely performed despite its huge potential in areas of complex anatomy or with rapid changes in radiotracer distribution, such as the renal tract [7]. “Total-body” PET/CT that would emulate routine acquisition of the type required for oncological imaging must, at least, encompass from the head to the pelvis, representing an ultra-extended FOV while still enabling movement of the patient into the scanner to acquire the legs if clinically indicated. This goal motivated the design of the Biograph Vision Quadra (Siemens Healthineers). In a research setting, the University of Pennsylvania group of Joel Karp has also developed a similar device called the PennPET Explorer [8]. Such scanners offer exciting opportunities for both routine clinical practice and research. *Cancer Imaging* has recently launched a series where key opinion leaders in the field will detail the initial experience and future directions in the use of these ultra-extended FOV scanners [9].

For those wanting to upgrade their existing PET/CT instrumentation or to enter, for the first time, into the exciting new-world of molecular imaging using an ultra-extended FOV PET/CT, there are many things to be considered. Having recently embarked on process of installing such a scanner, and despite having been involved in PET for more than 30 years and having designed several molecular imaging facilities, the author has been surprised by the challenges posed by this complex instrumentation. Accordingly, the goal of this article is to provide guidance to groups planning to embark on installation of ultra-extended FOV PET/CT scanners. While there are practical issues in the formative phases of project development, there are also decisions that need to be made about how this technology will impact departmental workflows, economics, and research agendas in coming years.

It is not the intention of the author to guide the choice of scanner, given that there are likely to be ongoing technical advances in the performance of these devices and their technical configurations. Rather, the aim is to guide project teams on the information they should seek from potential vendors and consider internally. As with all equipment purchases, the technical specifications of the systems are a critical factor in guiding purchases. System sensitivity, resolution and time-of-flight capability combined with software solutions offered by the vendor to enhance image quality or quantification should be carefully evaluated. Qualified medical physicists are vital to this process.

A short-list of the challenges in driving implementation of an ultra-extended FOV PET/CT installation are detailed below.

## Challenge #1- Financing

The first question an organization must ask itself is whether it can afford this technology.

There are currently 2 instruments that are available commercially that apply the principles of ultra-extended FOV PET/CT imaging; the µExplorer (United Imaging, China), which has an axial FOV of just under 2 m, and the Biograph Vision Quadra (Siemens Healthineers, USA), which has an axial FOV of just over 1 m. Enthusiasm for these devices is immense in the nuclear medicine community with many world-leading institutions rushing to install these scanners, creating supply chain challenges for the companies that manufacture them. The combination of these factors, along with the high complexity of the scanners and sheer volume of materials used to make them, means that these devices come at a premium price. They are more than twice as expensive as the next-best standard FOV digital PET/CT, and closer to 3–4 times the price of most of the installed base of analog PET/CTs in clinical use globally. Would it not be more cost-effective to just buy 2 or 3 conventional PET/CT devices?

Arguments against investing in more expensive technology were similarly raised at the time that PET/CT scanners first became available. When the Peter MacCallum Cancer Centre installed the 4^th^ PET/CT in the world in 2001, there were radiologists in the department who questioned the rationality of having an expensive CT added to an already expensive PET scanner that had limited throughput compared to a stand-alone CT. Therein was the very answer. The comparison ought not to have been against the throughput of CT but rather against the amortization of the more expensive component of the scanner, the PET device. The replacement of transmission scanning by rapid CT for the purposes of attenuation correction effectively doubled patient throughput, not to mention the clinical advantages of anatomical co-registration for correlative purposes. Provided that there were enough cases to augment throughput, the capital costs of the equipment per scan were the same or less than for a stand-alone PET. In addition to direct capital costs, more expensive and complex technology also costs more to service and maintain. Here, again, amortization of costs becomes a critical factor.

The financial case for ultra-extended FOV PET/CT must lie, at least partially, in greater throughput. This is made possible by the very substantial gain in sensitivity provided by greater coverage of the body by detector material. For example, the Siemens Biograph Vision Quadra has been shown to be 8–tenfold more sensitive, depending on the radionuclide, than the Biograph Vision 600, a standard FOV PET/CT with otherwise identical digital detector technology with time-of-flight capability [10]. Without changing the administered activity, equivalent signal-to-noise ratios, which are critical to lesion contrast and therefore detectability, can be achieved in imaging from head to pelvis in 2 min or less. Accounting for time to get patients on and off the scanner bed, 6 patients per hour is feasible with upwards of 40 patients per day possible. For standard photomultiplier tube PET/CT scanners, acquisition times can significantly exceed 30 min and, allowing for patient transfers, a daily throughput of more than 12–15 patients can be challenging. Standard FOV digital PET/CT devices can shorten acquisition times through enhanced sensitivity but 3 per hour would be a typical throughput.

This isn’t, however, the only economy that can be achieved. Leveraging this greater sensitivity can also allow a reduction in the administered activity required to acquire high-quality scans if acquisition times are less dramatically reduced. For expensive radiopharmaceuticals, particularly if produced at relatively low yields, this could represent significant cost savings. Consider, for example, radiopharmaceuticals produced from gallium-68 generators that progressively produce less activity due to the decay of the parent radionuclide, germanium-68. While sufficient activity may be available from a single synthesis to scan 4–5 patients when the generator is new, by the end of its life, only 1–2 cases may be possible. Reducing the activity by half and the scanning time by half, comparable image quality could be obtained, while amortizing the production and generator costs to a greater extent. The shorter the physical half-life of a tracer, the greater the potential benefits of more rapid scanning and a lower administered activity for patient in terms of radiation exposure. These scanners potentially bring carbon-11 radiopharmaceuticals for which the 20-min half-life is clinically impractical back into relevance if administered activity remains unchanged. Conversely, long-lived radionuclides, like zirconium-89, for which the administered activity is often reduced to limit radiation dose to patients, long scanning times can be significantly reduced. There is also the possibility of doing very late imaging, which has potential advantages for monoclonal antibodies with slow blood-pool clearance [11]. The potential benefits of such scanners to improving the efficiency and feasibility of clinical use of radiotracers that are currently constrained by low production yields or a short half-life is discussed in detail in an earlier review in this series. In a new department, a conscious decision to limit administered activity to patients can also significantly reduce the cost of lead shielding for uptake rooms (see below).

Importantly, relatively fixed costs of running a department include those of maintaining clinical staffing. In many jurisdictions, radiation exposure for staff is strictly controlled and limits the number of patients that a nuclear medicine technologist or nurse can manage per day. Reducing the administered activity to patients can allow greater productivity of these important and often limited personnel. With the rapid potential throughput of these scanners, the rate-limiting resource may become the reporting clinician who may struggle to keep pace with the scans coming off the device.

In developing business models, the capital costs must be balanced against careful considerations of the potential case mix, as well as fixed and variable operating costs. Of course, in some settings, demand may be limited by reimbursement restrictions or staffing levels and such scanners are unlikely to be financially viable without either capital equipment grants from government agencies or philanthropic organizations that recognize the clinical and research opportunities that these devices provide.

Industry-funded research is a further source of operational revenue and ability to do the same workload in a shorter interval potentially opens greater capacity to accommodate research studies in a busy department.

## Challenge #2- Space

This has two aspects. Firstly, the scanner itself may require a substantially larger room than a conventional PET/CT in the case of the µExplorer but not for the Biograph Vision Quadra, which has essentially the same longitudinal and width footprint as its standard FOV equivalent. Both scanners require rather large equipment rooms to house the electronic cabinets and computers necessary to power and process scans. Secondly, higher throughput potentially necessitates more uptake rooms. Retrofitting existing departments to accommodate the higher throughput made possible by these scanners may not be feasible without significant expense and should be considered as part of project planning. Approximately 6 uptake rooms are required to efficiently utilize the throughput capability of ultra-extended FOV PET/CT scanners.

## Challenge #3- Structural engineering

**Fig. 1:**
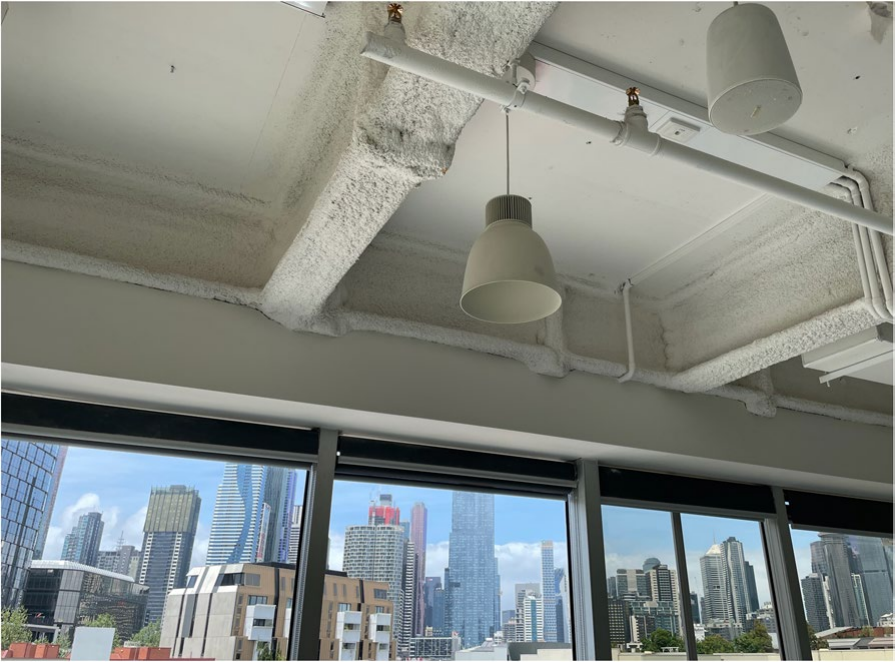
Structural steel was required to strengthen the concrete slab. Access to the floor below the scanner needed to be negotiated with the tenant. Despite the increased cost, patient, and staff amenity of a department with access to natural light, not to mention views is considered to be an advantage of locating imaging departments above ground rather than their traditional basement positioning

Current devices are 1.5–3 times heavier than existing PET/CT devices. Combined with the need to create floor channels to convey cabling and chilled water into the gantry, this may challenge the integrity of floor slabs rated only for conventional loads. This becomes particularly relevant for departments that are elevated off ground level to any degree. While placement in the lowest basement level is often a response to this limitation, the lack of natural light tends to reduce patient and staff amenity. For our new facility, which is located on the 8^th^ floor, substantial structural steel reinforcing was required, but the views over the city from the scanner and uptake rooms made this a worthwhile investment (Fig. 1).

## Challenge #4- Power supply

These are complex scanners with substantial computer processing requirements and therefore draw significant amounts of power, which needs to be very stable. It is important to involve an electrical engineer early in the process to determine whether upgrading of electrical supply systems may be required in addition to the dedicated power cabinet required for these systems. Depending on the stability of power supply, uninterruptable power suppler (UPS) devices may also be worthwhile. Periodic power testing with switch-over to generator power can be problematic and should be scheduled out-of-hours. Recalibration will be required after such testing and should be coordinated with the facility operator.

## Challenge #5- Managing heat load

The components of the PET scanner and operation of the CT generate a significant heat load. Furthermore, the detectors in the former require a tightly controlled environment in terms of both temperature range and fluctuation as well as controlled humidity. Accordingly, high capacity and potentially redundant air-conditioning is necessary in both the scanner and equipment rooms. The current scanners also require chilled water. The location of chillers or tapping into existing chilled water supplies should be considered early in project management and costed into the capital budget. Generator back-up of the air-conditioning systems to the scanner and equipment room and to the chillers is a worthwhile investment, particularly in parts of the world with more extreme climates or unreliable power supplies.

## Challenge #6- Data handling

Although extended FOV PET/CT allows dynamic imaging of all the major organ systems simultaneously, the list mode acquisition involved generates vast amounts of data. This can amount to a terabyte per hour or more in high count studies. Even static images are significantly larger than when acquired on a conventional PET/CT. Many early adopters of this technology found that they quickly filled the memory provided on the systems and have had to retrofit high-capacity, typically several petabytes, storage-arrays. Strategic decisions are required as to whether raw data is stored for future analysis or only the DICOM-compliant reconstructed images, which can be stored on institutional or cloud-based PACS solutions. Transfer of large datasets from the processing computer to the storage device and retrieval from it requires high data transfer speed, for which fiberoptic connections are superior to copper wire. With increasing use of artificial intelligence to analyze these complex studies and evolution of the algorithms, storage of raw data is advisable for future analysis, particularly for research studies. Automated segmentation approaches are likely to benefit quantitative analysis of PET data. The computational power required for such AI algorithms needs access to high-end workstations with multiple CPUs and GPUs. Cloud computing is also an option that can be considered for advanced users. In a world where cybersecurity threats are ever-present and increasing, patient privacy and research data protection is a vital consideration. Accordingly, in addition to requiring appropriate levels of data security from the PET/CT supplier, engagement of independent information technology expertise is strongly recommended.

## Challenge #7- Radiation safety and radiopharmaceutical procurement

It is important to open an early discussion with the radiation safety regulators in your jurisdiction as the cost of lead shielding and other protections for workers in the department need to be factored into the project planning. Because of the substantially higher sensitivity of ultra-extended FOV PET/CT scanners, dramatically higher throughput can be achieved if administered activity remains unchanged compared to standard FOV devices. This will add to the potential total radiation burden to staff working in the facility. Multi-scanner departments that wish to add a new ultra-extended FOV PET/CT into existing workflows may prefer to have a single protocol for drawing up and administration of radiotracers and to only alter the acquisition time depending on the scanner to which the patient is assigned.

However, there is also an opportunity to significantly reduce administered activity while still decreasing scan acquisition time to a degree. The lower administered activity to any given patient potentially allows a lower thickness of lead shielding to meet prescribed radiation dose limits based on staff and public occupancy of adjacent areas. This can reduce both the cost and structural engineering costs associated with a new project but could limit future flexibility in administering higher activities to patients.

If radiopharmaceuticals are produced onsite, higher throughput combined with reduced administered activity can reduce the cost per patient scanned from a production run by amortizing kit or synthesis cassette, radiochemist, and quality assurance costs. Reducing the number of cyclotron-runs could be particularly advantageous for engineering staff requirements. If purchased from an external GMP supplier, it will depend on whether the supply is costed by the delivered activity, wherein costs could be significantly reduced, or by individual patient doses, in which case it may be possible to negotiate a reduced cost for a lower activity in each syringe. Because scan acquisition times are reduced, patients receiving short-lived radiopharmaceuticals can be batched to more efficiently to amortize production and quality-assurance costs.

## Challenge #8- Staffing

In many parts of the world, the growing popularity of molecular imaging and most particularly in PET/CT has created a shortage of trained nuclear medicine technologists (NMTs). This, combined with the increased throughput capacity of modern scanners, means that NMTs may need to supervise more patients in an average working day and, in some cases, contribute to significantly increased occupational radiation exposure. A potential advantage of reducing both administered activity and acquisition times is that greater productivity can be achieved from a scarce NMT workforce. Clinical assistants and cannulation by phlebotomists to aid patient handling can further increase departmental efficiency relieving NMTs of these responsibilities.

Subject to the speed with which physicians or radiologists can report scans, nuclear medicine specialists may become the rate-limiting factor to increased throughput capacity. However, more efficient clinician time-management could also be achieved.

Higher departmental throughput could also improve the efficient use of administrative and nursing staff, recognizing that these valuable team members must not feel overwhelmed by the pressure of an extremely high throughput workplace.

Conversely, the complexity of advanced quality assurance protocols and advanced processing algorithms required to fully leverage the technical capabilities of these scanners may require additional medical physics personnel. Retraining on image interpretation may be valuable, for even experienced PET/CT readers and the younger generation of molecular imaging specialists who have never learned the principles of quantitative PET compartmental modeling may need further training in these techniques.

## Challenge #9- Leveraging technical advantages to adapt workflows and acquisition protocols

As noted above, decisions regarding whether to decrease administered activity, scan acquisition duration, or a combination of both are important for delayed “total-body” imaging protocols and may depend on whether the scanner is assigned simply another scanner in a multi-scanner department, or as a stand-alone device.

The unique capability of these scanners to perform “all organ” dynamic imaging could provide large amount of clinically relevant data include first-pass cardiac ejection fractions, pulmonary perfusion, renal perfusion and excretion of relevant tracers, arterial phase perfusion of structural abnormalities and as an input function for quantitative kinetic studies [12], and early blood pool imaging. While vendors are involved in research to develop “out-of-the-box” solutions, departments are likely to benefit from “in-house” capability.

It is important, in the author’s opinion, not to consider this as simply a PET/CT capable of faster acquisition. The images provide superior contrast to most scanners and may require clinicians to re-establish the sensitivity with which they report subtle findings in order not to increase false-positive results. Similarly, quantitative thresholds and reconstruction algorithms that have been set on conventional scanners may need to be adapted for these scanners. The physics community are actively addressing these issues [13, 14].

The possibility of “all organ” dynamic imaging offers new frontiers in understanding the interaction of organs as an application of systems biology [15].

## Challenge #10- Marketing to other clinical specialists and patients

Although potentially an overstated and emotive rather than scientifically-based concern, radiation exposure from diagnostic imaging is often given as a reason to limit or omit use of PET/CT in situations where it may significantly improve the accuracy of diagnosis with potential prognostic and therapeutic consequences. The use of this imaging for assessment of suspected maternal malignancy during pregnancy is one such example [16]. Others include paediatric malignancy and the screening of patients with a high-lifetime risk of malignancy due to carriage of germ-line mutations, particularly those, such as *BRCA* and *Tp53* mutations, that might also predispose to defective DNA-repair. Guidelines for screening at-risk populations, such as those for succinate dehydrogenase (SDHX) germ-line mutation carriers that recommend from an early age [17], are beginning to recognize the superior diagnostic accuracy of PET. The ability to use extremely low administered activity [14] in these situations may increase physician and patient acceptance of scans on ultra-extended FOV scanners and provide a marketing opportunity. Young patients with chronic rheumatologic conditions that might require serial monitoring are another potential population that could benefit from lower administered activity.

The combination of lower radiation dose and faster scanning provides greater patient comfort, particularly for patients with pain or other difficulties lying for prolonged periods. Although it might be thought that patients with claustrophobia might find the long bore-length daunting, the ability to do rapid scanning and because the head can be positioned at to edge of the scanner, many patients are reported (Personal communication with University of Groningen PET facility) to prefer the ultra-extended FOV PET/CT to standard FOV scanners. Our own early experience supports this impression with patients finding the short acquisition times tolerable without need for sedation.

A potential marketing advantage of these scanners is the dramatically increased signal to noise ratios (SNRs), which can improve lesion contrast [10] and give aesthetically impressive image quality as demonstrated in other articles in this series. For many tracers, SNRs in tumour sites increase with time but, due to radioactive decay, image quality tends to reduce due to lower counts being available. The dramatically superior sensitivity of ultra-extended FOV PET/CT will provide higher image quality at late time-points and corresponding improved lesion contrast. As is commonly said, a picture is worth a thousand words. When clinicians see the images acquired on these scanners, referral patterns may well favour sites that take the risk of installing these high-end imaging devices. Semi-quantitative imaging parameters consistent with standard FOV cameras are achievable with a ten-fold reduction in administered activity or reduction in scanning time [13] but the ability to provide fully quantitative data from dynamic imaging will provide opportunities for a much deeper level of PET reporting. The attraction of working with the latest technology may also be helpful in recruiting or retaining staff, particularly nuclear medicine technologists and medical physicists.

The unique capabilities of ultra-extended FOV PET/CT scanners will provide pharmaceutical and radiopharmaceutical companies unique information in the development of their products and will provide a potential source of revenue to offset the higher capital cost of these scanners.

## Conclusion

Molecular imaging represents a powerful exemplar of what is known as “convergence science”, bringing together, as it does, physics, chemistry, engineering, biology, software design and clinical medicine. The teamwork that is required to run an efficient and effective PET service needs to be replicated in planning, installing, and running an ultra-extended FOV PET/CT. Involvement of key stakeholders and the content experts required to meet the challenges detailed above and high-level business-model planning are vital to a successful project. In the beginning, it will seem like a risky venture, but the author is confident that this is the future of PET/CT.

“Why not go out on a limb? Isn’t that where the fruit is?”

- Frank Scully, American writer

## Data Availability

No relevant data or material included.

## Abbreviations

CPUs: Central processing units
FOV: Field-of-view
GMP: Good manufacturing practice
GPUs: Graphics processing units
NMTs: Nuclear medicine technologists
PET/CT: Positron emission tomography/computed tomography
SDHX: Succinate dehydrogenase mutation carrier
SNRs: Signal to noise ratios
UPS: Uninterruptable power supply
